# Supplementary material for: Are carbon emissions trading and green financial instruments synergistic? -Comprehensive quantitative research based on content analysis
Source: PLoS One. 2024 Mar 7;19(3):e0298601. doi: 10.1371/journal.pone.0298601 (PMC10919601; doi:10.1371/journal.pone.0298601)
Supplement: S1 Data — (ZIP) [file pone.0298601.s001.zip › Data/地级市Z_F工作报告.zip!/╦─┤¿/└╓╔╜╩╨/└┤╫╘╣1⁄2╓┌║┼í╛┬φ┐╦╩2╛▌═°í┐_2016.docx]

各位代表：

现在，我代表乐山市人民政府，向大会报告工作，请予审查，并请市政协各位委员提出意见。

“十二五”及2015年工作回顾

“十二五”时期的五年，是我市发展中很不平凡的五年。五年来，我们面对错综复杂的宏观形势和改革转型的繁重任务，全面贯彻落实党的十八大、十八届三中、四中、五中全会和习近平总书记系列重要讲话精神，深刻把握“四个全面”战略布局，始终保持定力谋发展、攻坚克难抓落实，较好地实现了“十二五”规划目标任务，经济社会发展取得了新的重大成就，为全面建成小康社会奠定了坚实基础。

这五年，是经济保持较快增长、综合实力显著增强的五年。我们主动把握和适应经济发展新常态，精准施策、多点发力顶住经济下行压力，确保了经济增长换挡不失速。五年来，全市地区生产总值年均增长11．3％，分别快于全国、全省平均增速3．5个、0．5个百分点，“十二五”末经济总量是“十一五”末的1．8倍。2012年，全市经济总量突破千亿元大关，成为全省第8个“千亿俱乐部”成员；6个县（市、区）经济总量迈上百亿元台阶。全市人均地区生产总值提高到39973元、是2010年的1．8倍，与全国平均水平的比例差距缩小6．5个百分点左右。

这五年，是发展方式加快转变、质量效益稳步提升的五年。我们始终保持转型发展战略定力，坚定不移转方式调结构，推动形成结构优化、动力转换、提质增效的良好态势。三次产业结构由13．4：59．5：27．1调整为11：58．9：30．1，第三产业比重上升3个百分点。新兴产业主营业务收入占规模以上工业比重提高15个百分点，高新技术产业总产值占规模以上工业比重提高24个百分点。单位地区生产总值能耗累计下降27．54％。科技进步对经济增长的贡献率提高5个百分点，乐山高新区升级为国家级高新区。五年累计完成地方一般公共预算收入370亿元、是“十一五”总量的2．5倍，其中税收收入233．8亿元、是“十一五”总量的2．1倍。

这五年，是基础建设强力推进、城乡面貌日新月异的五年。我们坚持以项目为支撑，实施了一批事关发展全局的重大项目，累计完成全社会固定资产投资3829亿元，是“十一五”总量的2．5倍。交通、水利、能源、信息等基础设施水平全面跃升，乐雅高速、乐自高速、成绵乐高铁先后通车，乐山迈入高铁时代。新区建设和旧城改造同步发力，岷江东岸、青江新区初具规模，城市建成区面积由102．8平方公里扩展至139．7平方公里，城镇化率提高7．8个百分点、达47．3％。城乡统筹协调并进，建成幸福美丽新村717个，彝家新寨261个、覆盖彝区70％的行政村。

这五年，是主动融入国家战略、开放合作纵深拓展的五年。我们抢抓开放布局调整新机遇，以充分开放合作赢得发展先机。积极搭建旅博会、陶博会、不锈钢产品交易博览会等开放合作平台，旅博会和与亚太旅游协会联办的峨眉高峰论坛永久落户乐山。招商引资成效显著，港中旅、中科实业、中国建材、中国华录、中信国安、万达集团等一批知名央企民企来乐投资兴业，累计引进到位市外内资2561．6亿元，是“十一五”总量的2．8倍。全市国内外友好城市增至36个，建立友好交往和贸易往来关系的国家和地区达109个，“乐山造”产品出口国家和地区达34个。

这五年，是人民生活持续改善、社会事业全面进步的五年。我们坚持把人民对美好生活的向往作为奋斗目标，重民生、补短板，让全市人民有更多获得感。城乡居民家庭财产普遍增加，城乡居民人均可支配收入分别是“十一五”末的1．8倍、1．9倍。群众生活水平不断提高，消费结构演进升级，城乡居民家庭恩格尔系数分别下降4．5个、6．3个百分点。覆盖城乡的社会保障制度更加完善，保障待遇水平逐年提高，建成城镇保障性住房4．6万套，新改建农村住房5．7万户。全市贫困发生率从19．5％下降到4．2％。连续五届获全国科技进步先进市称号，城乡免费义务教育全面实现，卫生计生服务水平显著提升，各项社会事业长足发展。

刚刚过去的2015年，我们在市委坚强领导下，组织动员全市广大干部群众，紧紧围绕“转型升级年”工作主题，统筹稳增长、调结构、促改革、惠民生、防风险，各项工作取得了新的成绩，实现了“十二五”圆满收官。全市实现地区生产总值1301．2亿元、增长9．1％，地方一般公共预算收入85．5亿元、增长8．6％，城镇居民人均可支配收入26361元、增长8．7％，农村居民人均可支配收入11649元、增长9．9％。

一、稳定增势取得新成效

项目投资拉动有力。完成全社会固定资产投资987．8亿元、增长14．3％。新万兴首批飞机专用碳纤维复合材料制品、嘉洋科技GPP芯片一期、市公交客运总站等项目竣工，万达广场、中信国安峨眉山城、成贵铁路、成昆铁路成峨段扩能工程等项目加快建设，岷江航电犍为枢纽、峨眉山国际旅游度假区旅游项目、连乐铁路等项目落地开工，港中旅集团乐山旅游资源整体开发、峨汉高速、德国EPC公司高性能碳纤维等项目成功签约。积极争取中央专项建设基金，32个项目获37．2亿元基金支持。率先在全省举办政府和社会资本合作（PPP）项目推介会，激发社会资本活力，完成民间投资659．8亿元、增长10．2％。

实体经济回暖向好。实行规模以上企业“一企一策”帮扶全覆盖，规模以上工业增加值增长11％、较上年提高4．9个百分点，规模以上工业企业净增15家、达629家。设立应急转贷专项资金，组建上市和挂牌企业后备资源库，4家企业登陆“新三板”。积极争取直购电、富余电量、“水火置换”和直供气等优惠政策，减轻企业生产成本15．7亿元。加大市场拓展力度，成功举办首届陶博会、不锈钢产品交易博览会，规模企业产销率回升至95．4％、较年初提高2．8个百分点。

消费市场繁荣活跃。实现社会消费品零售总额552亿元、增长13．3％。电子商务快速发展，创建全国电子商务进农村示范县1个，电商交易额、网络零售额分别增长34．5％和45．4％。信息消费加快发展，宽带网络提速降费、4G网络覆盖、高清电视普及等工程加快实施，成功列入国家智慧城市试点，建成全省首批“全光网城市”。积极落实楼市新政，商品住房销售面积增长28％。

二、产业转型迈出新步伐

服务业发展质量提高。规模以上服务业企业净增73家、达154家，服务业增加值达391．7亿元。全域旅游乘势发展，累计接待国内外游客3920．9万人次、增长16．9％，实现旅游综合收入499．2亿元、增长29．4％，国内外游客在乐平均停留天数达1．6天。成功组织承办第二届旅博会、亚太旅游协会2015年会、环中国国际公路自行车赛、四川国际峨眉武术节、全国桥牌A类俱乐部联赛、黑竹沟户外障碍挑战赛等重大活动和赛事，PATA—乐山山地旅游示范基地落户，桃源山居晋升4A级景区。金融业加快发展，重庆银行入驻乐山，县级农村信用联社改制组建工作进展顺利，全市银行业金融机构本外币各项贷款余额1238．3亿元、较年初增长13．8％。商贸物流稳步发展，王府井百货建成开业，西部不锈钢金属综合交易市场建成运营。

工业结构加快调整。新兴产业主营业务收入占规模以上工业比重提高3个百分点、达38％。净增国家高新技术企业14家、达70家。传统产业改造升级，完成技改投资238．4亿元、增长10％。轻重工业比重调整为29．6：70．4，轻工业比重上升1．3个百分点。加快淘汰落后产能，关闭小煤矿7家，其亚电解铝生产线全部关停，单位工业增加值能耗下降9．22％。园区集聚功能增强，工业集中度提高1个百分点、达73％。

现代农业稳步发展。全年粮食总产109．2万吨。新建现代农业万亩示范区12个、达51个。新发展农民专业合作社430个、家庭农场417家、市级以上龙头企业6家，农业产业化经营带动面提高到69％。我市获批全国农民专业合作社贷款担保补贴试点，新创建第三批国家现代农业示范县1个。有机生态农业加快发展，“三品一标”农产品总数达706个，创建省级有机产品示范县1个。农村发展条件不断改善，新建成幸福美丽新村200个，新（改）建县乡公路180公里、通村公路608公里，改造升级农村电网900公里，解决30．7万农村居民饮水安全问题。

三、改革创新迸发新活力

深化改革蹄疾步稳。出台37个专项改革方案，重点改革稳步推进。我市成为首批国家级旅游业改革创新先行区、全省首个旅游综合改革试点市。建立城乡统一户口登记制度，发放居住证6．3万本。农村土地承包经营权确权登记颁证工作基本完成，新增土地流转面积16．9万亩、达53万亩。“三证合一、一照一码”登记制度全面实行，新登记市场主体2万个、增长14．5％。在全省率先建成市场主体信用信息交换中心。国资国企改革不断深化，2个外派监事会进驻17家市属国企开展工作。

创新创业有力开展。科技进步对经济增长的贡献率提高1个百分点、达50％。专利申请突破1400件，创历史新高，专利成果新实施项目392项、新增产值93．8亿元。大众创业、万众创新深入推进，成立乐山中科创新育成中心，建成大学生创业孵化园等各类创业园区25个。市科技企业孵化器大平台、峨眉创客梦工厂创新创业孵化器列入省重点科技平台建设计划，竹类生物技术与资源开发实验室成为我市首个省级重点实验室。圆满完成第三届科博会成都经济区轮值市参会参展工作。

对外开放水平提升。多层次推进开放合作，新签约各类战略合作协议18个。组团参加中外知名企业四川行、中国西部（四川）进口展暨国际投资大会等活动，精准对接目标企业，全年新签约正式合同招商项目79个、投资总额773．1亿元。引进到位市外内资560．6亿元，实际利用外资1．2亿美元。乐山制造、乐山服务“一体化”走出去步伐加快，新增进出口实绩企业12家、达77家，新增境外投资项目5个、对外投资总额3．9亿美元。

四、群众生活得到新改善

精准脱贫全面发力。小凉山—乌蒙山乐山片区和市列重点区域脱贫攻坚统筹推进，投入各类扶贫资金18．7亿元，新建成扶贫新村16个、彝家新寨33个，精准减贫4．5万人。完成精准识别建档立卡工作，实现贫困村驻村帮扶全覆盖。创新实施彝区“七个一”工程，新建幼儿园88所、村卫生室110个。向彝区定向培养中专医学生200人，向“三县一区”选派支教教师160人。促进彝区农村劳动力转移就业11万人，彝绣等产业带动彝区妇女就业4．6万人。

民生保障得到加强。十项民生工程和20件民生实事全面完成。完成大学生创新创业培训8520人，城镇新增就业4．9万人。企业退休人员养老金待遇、失业保险金待遇、最低工资标准、城乡低保标准和农村五保供养标准调整提高，城乡居民基本养老保险和医疗保险参保率分别达95％、98％。物价保持基本稳定，主城区居民生活用水和天然气实行阶梯价格制度。深入推进“百万安居工程建设行动”，保障性安居工程住房新开工9887套、竣工8856套。养老服务体系加快建设。“量体裁衣”式残疾人服务模式全面推广。市慈善总会荣获全国先进社会组织。

各项事业协调发展。乐山职业技术学院新校区、乐师附小新改建等项目有序推进，2个区县通过义务教育基本均衡国家督导评估验收，教育助学工程惠及40．2万人次。县级公立医院综合改革稳步推进，市精神卫生中心迁建工程完工，民营医疗机构新增29家。计划生育政策平稳过渡，人口自然增长率2．31‰。成功申报创建国家公共文化服务体系示范区。直播卫星户户通和应急广播工程启用。首届老年人运动会顺利举办。档案数字化建设水平得到提高。完成空白乡镇邮政局（所）补建。对口援藏工作扎实开展。民族、宗教、妇儿、外侨、对台、保密、人防、气象等工作取得新成绩。

五、发展环境呈现新气象

生态环境持续改善。强化国土空间用途管制，基本完成永久性基本农田划定。综合治理水土流失面积224．2平方公里，巩固退耕还林面积92．2万亩，森林覆盖率达55．47％。实施减排项目150个，中心城区空气质量持续保持优良水平，PM10年均浓度值比2013基准年下降20．8％。市第一污水处理厂扩建完工，小流域综合治理收效明显。新创建省级生态县（区）3个、生态乡镇6个。农村生活垃圾治理通过国家检查验收。

社会环境和谐安定。“六五”普法通过省级验收，新创建全国民主法治县2个、法治村2个，省级法治示范县1个。“亲情工作法”“阳光信访”有效开展，调处矛盾纠纷1．5万余起。反恐防暴全面加强，社会治安防控体系不断完善。成功组织省级防震救灾综合演练，应急处突能力得到提升。安全生产大检查大整治深入开展，“打非治违”专项行动收效明显，安全生产形势总体稳定。新创建国家级安全社区1个、省级8个。有力有序开展金口河“1．14”地震抗震救灾工作。完成食品安全检（监）测能力建设。优抚安置政策得到落实，我市蝉联“省双拥模范城”称号。

政务环境不断优化。简政放权、放管结合、优化服务力度加大，公布市级行政权力清单和公共资源交易目录，取消和调整非行政许可审批项目37项。扎实开展“三严三实”专题教育，从严落实党纪党规和“两个责任”，严肃查处了一批发生在群众身边的“四风”问题。依法规范决策程序，开展重大决策合法性审查160件、社会稳定风险评估71件。主动接受人大、政协和社会各界监督，认真落实人大代表建议和政协委员提案办理制度，办结市人大代表建议、市政协委员提案共413件，满意和基本满意率100％。

各位代表：“十二五”及2015年经济社会发展取得的成绩来之不易，为开启“十三五”发展大幕夯实了基础、积蓄了后劲。这是市委坚强领导的结果，是市人大、市政协和社会各界监督、支持的结果，是全市广大干部群众共同奋斗的结果。我代表市人民政府，向全市各族人民，向驻乐部队指战员、武警消防官兵、政法干警，向各民主党派、工商联、无党派人士、各人民团体和各界朋友表示衷心的感谢，并致以崇高的敬意！

同时，我们也清醒地看到，全市经济社会发展仍然面临不少的困难和挑战，主要表现为：经济下行压力依然较大，回暖向好的基础尚不稳固；结构调整任务艰巨，创新能力总体不足；城乡发展不够平衡，资源环境约束进一步加剧；公共产品特别是优质公共服务资源供给还有短板，部分群众生活还比较困难；法治政府和服务型政府建设还需加强等等。对这些困难问题，我们将高度重视，采取措施，认真解决。

“十三五”时期主要考虑

各位代表：“十三五”发展征程已经开启。今后五年，是乐山全面建成小康社会的决胜时期，是全面深化改革的攻坚时期，是推进“转型升级、美丽发展”的关键时期。这期间，随着国家“一带一路”、长江经济带、新一轮西部大开发、全省“三大发展战略”的全面实施，“中国制造2025”“互联网＋”行动计划、全省全面创新改革驱动转型发展等系列重大部署深入推进，为我们培育发展新动力、拓展发展新空间、厚植发展新优势，提供了难得的历史机遇。同时，宏观经济环境仍然存在一些不确定因素，我市“实力不强、结构不优、贫困不少、发展不平衡”的基本市情还没有根本改变，经济社会发展还面临不少挑战。但总体上看，经济发展向好的基本面没有变，持续增长的支撑基础和条件没有变，结构调整优化的前进态势没有变，经济发展有很大的潜力、韧性和回旋余地。只要我们认清大势、保持定力、干在实处，就一定能掌握发展主动权，保持发展好势头，开拓发展新境界，实现总体小康向全面小康的历史跨越。

市委六届九次全会审议通过了《关于制定乐山市国民经济和社会发展第十三个五年规划的建议》，为我市未来五年的发展指明了方向，描绘了蓝图。市政府根据《建议》组织起草了《乐山市国民经济和社会发展第十三个五年规划纲要（草案）》，已印发大会审议。这里，就相关内容作简要说明。

关于发展定位。当前和今后一段时期，乐山发展的总体战略定位是，建设国际旅游目的地、全省高新技术产业增长极、四川综合交通次枢纽和大小凉山脱贫攻坚示范区。这“四个发展定位”充分彰显了创新、协调、绿色、开放、共享“五大发展理念”，集中体现了乐山“转型升级、美丽发展”的主题，是通过长期探索对乐山发展方向的高度概括，经过实践检验，干部群众广泛认同。“四个发展定位”是一个整体，相互联系、互为支撑。

关于发展目标。“十三五”时期，全市经济社会发展的总体目标是，保持发展速度高于全省平均水平，全市地区生产总值、人均地区生产总值、城乡居民人均可支配收入在2010年基础上实现翻番，力争地区生产总值迈上2000亿元台阶，力争城乡居民人均可支配收入达到全国平均水平，先于全省全面建成小康社会。上述总体目标的制定，既充分尊重全市人民的发展愿望，又充分考虑了宏观环境和支撑条件，体现了自我加压、跳起摸高。只要我们保持定力、创新实干，这一宏伟目标就一定能够实现。

关于发展路径。未来五年，我们将突出“六条发展路径”。一是旅游“国际化”。围绕国际旅游目的地建设，统筹发展全域旅游，到2020年，全市旅游综合收入突破900亿元，过夜游客平均停留天数达到2．5天以上。二是工业“高端化”。坚定不移走新型工业化路子，建设现代工业强市，到2020年，新兴产业产值占规模以上工业的45％以上。三是农业“有机化”。坚持生态型道路、有机化方向、现代化目标，不断增强农业可持续发展能力，力争有机农产品认证数量年均增长30％，到2020年农业产业化经营带动面达到75％。四是景城“一体化”。优化城市生态、生产、生活空间布局，推动景城一体、产城一体、城乡一体协调发展，到2020年，城镇化率达到55％左右。五是交通“枢纽化”。加快重大交通项目建设，到2020年，乐山军民合用机场建成运营，岷江港航电综合开发工程取得突破，基本实现县县通高速，形成公路、铁路、航空、水运立体综合交通运输体系，基本建成四通八达、内外互联互通、立体交叉、网状发展的综合交通次枢纽。六是扶贫“精准化”。把脱贫攻坚作为“十三五”期间的头等大事，全力推进精准扶贫、精准脱贫，到2019年，所有贫困县（区）、贫困村全部摘帽，建档立卡贫困人口全部脱贫。

2016年工作安排

2016年是实施“十三五”规划、全面建成小康社会决胜阶段的开局之年。市政府工作的总体要求是：牢固树立创新、协调、绿色、开放、共享发展理念，坚持稳中求进工作总基调，紧扣“四个发展定位”，以“创新投资年”为主题，突出全面创新改革牵引，着力稳定发展增势、转变发展方式、培育发展动能、提高发展质量、突出发展惠民，全力促进经济社会持续健康发展，确保“十三五”开好局起好步。主要目标是：地区生产总值增长8％，全社会固定资产投资增长11％，规模以上工业增加值增长8．5％，社会消费品零售总额增长11％，地方一般公共预算收入增长5％，城镇居民人均可支配收入增长8％，农村居民人均可支配收入增长9％，全面完成省下达各项目标任务。重点抓好以下工作：

一、坚持创新引领，着力强化转型发展支撑

突出“创新投资年”工作主题，以全面创新改革为契机，加大投资促进力度，为乐山稳增长、调结构、促转型提供强大动力。

深入实施创新驱动。依托四川推进全面创新改革试验区辐射带动作用，加快全域创新步伐，促进创新型城市、全省科技服务业发展重点市建设。突出企业创新主体地位，深入实施高新技术企业倍增行动、科技型中小微企业培育工程，力争新增国家高新技术企业5家，建成在孵企业或项目1000个以上。打造创新平台载体，加快建设一批科技孵化器、重点实验室和产业技术创新联盟，推进乐山国检中心建设，组建北大乐山科技园。完善政产学研用协同创新体系，支持在乐高校、科研院所就地整合创新资源、开展技术攻关、转化科技成果。积极培育军民融合产业，推进军民融合创新发展。深入实施大众创业、万众创新，支持建设“孵化＋创投”等新型孵化器，发展云端众创、众包、众扶、众筹等新模式。加大青年拔尖人才、紧缺人才、高技能人才培养力度，积极引进高层次人才、创新团队，激发各类人才创新活力。

突出项目投资拉动。发挥项目投资对稳增长、促转型的关键作用，加大对上争取、对内挖潜、对外引进力度，持续扩大有效投资，确保全社会固定资产投资突破1000亿元。加强要素保障和跟踪服务，突出抓好23个省列、182个市列重大项目建设，确保完成投资350亿元以上。抓紧启动建设一批标志性、产业化重大项目，重点推进岷江航电犍为枢纽建设，力争开工老木孔枢纽和龙溪口枢纽项目，做好东风岩枢纽和乐山港前期工作。全方位加强投资促进，提升招商引资质量和实效，力争全年引进到位市外内资500亿元。统筹用好专项建设、产业发展、新区建设、创新创业、基础设施建设等投资引导基金，通过推广PPP、政府购买公共服务、发行企业债券等方式，多渠道激发社会投资活力。

聚焦重点改革推动。紧扣“去产能、去库存、去杠杆、降成本、补短板”要求，加快推进供给侧改革，有效提升供给质量和效率，实现供需两端协同发力。深化农业农村改革，稳妥推进农村承包地经营权抵押贷款、土地流转收益保证贷款、农村产权抵押融资试点，完成农村小型水利工程管理体制改革，抓好国有林场林区改革和供销社综合改革试点。深化国资国企改革，提高国有资本投资运营效益。深化财税体制改革，转变财政支持发展方式，加快构建产业发展投资引导基金体系和财政金融互动政策体系。深化商事制度改革，加强事中事后监管。做好不动产统一登记。统筹抓好教育、文化、卫生等领域改革。

二、坚持文旅带动，着力建设国际旅游目的地

最大程度发挥文旅资源优势，加快打造乐山旅游“升级版”，不断提升乐山旅游的知名度和国际化水平。

发展全域旅游。抓好国家全域旅游示范区创建工作，推进旅游业全区域、全要素、全产业链发展。加快核心景区提质扩容，推进峨眉山景区应急救援疏散通道、零公里停车场改建、乐山大佛景区北门广场改造提升等项目，争创峨眉山国家旅游度假区和乐山大佛省级旅游度假区。加大旅游资源整合力度，抓好港中旅集团乐山旅游资源整体开发、嘉阳·桫椤湖、大风顶等项目建设，支持黑竹沟景区创建国家生态旅游示范区，新创国家A级景区3个。创新“旅游业＋”思路，促进旅游与文化、体育、商贸、农业、水利等融合发展，大力培育康养、休闲度假、文化创意等新业态。深挖佛禅文化、武术文化、民族民俗文化等内涵，加快黄湾武术文化小镇、智慧旅游文化创意产业园、阳花渡文化产业园等建设，促进文化旅游深度融合、共生共进。

创新体制机制。以国家级旅游业改革创新先行区、全省旅游综合改革试点市为平台，全面完成各项规定任务、商定任务和自选任务，探索形成具有乐山特色的旅游综合改革路径。深化旅游综合管理体制改革，理顺涉旅管理部门、景区管理单位职能职责，构建职责清晰、配合有效的旅游综合协调机制。完善旅游投融资机制，设立旅游文化产业发展基金，搭建旅游投融资平台，促进多种方式融资筹资。健全旅游市场监管体制，完善诚信乐山旅游信用公示与服务平台建设。推动集中行使相关处罚权，实行旅游综合行政执法，规范旅游市场秩序。

拓展旅游市场。创新市场化、网络化营销模式，提升营销实效，力争全年游客接待量和旅游综合收入分别增长10％和18％。精心组织承办好第三届旅博会、峨眉高峰论坛等重大会展活动，争取把旅博会打造成国际旅游业界品牌展会。不断深化与世界旅游组织、亚太旅游协会、国内外友好城市的国际旅游交流合作，提高乐山旅游经济外向度和国际旅游竞争力。着力拓展入境游市场，积极争取落地签、过境免签等政策，推行境外旅客购物离境退税，力争入境游人次增长15％。

三、坚持高端切入，着力做强工业经济

抓住重点项目、重点企业和重点产品，加快新兴产业培育和传统产业改造，打造四川高新技术产业增长极和传统产业转型升级示范市。

加快产业升级。聚焦战略性新兴产业和高端成长型产业，重点发展电子信息、清洁能源、新材料、现代装备制造、生物制药等五大新兴产业，确保新兴产业主营业务收入占规模以上工业的比重提高到39％。抓好嘉洋科技GPP芯片二期、新万兴碳纤维复合材料生产线扩能、和邦高性能碳纤维等项目，发展壮大电子信息、新材料等产业。加快华发纯电动汽车生产基地等项目建设，错位发展新能源汽车、现代装备制造等产业。支持新长征药业园抗生素、抗结核药等主导品种升级改造和技术创新，促进生物医药产业集群化发展。大力改造提升冶金建材、盐磷化工、农产品加工等传统产业，抓好华构住工新型建材、国家建筑陶瓷产品质检中心（四川）等建设，确保福华年产8万吨甘氨酸技改等项目竣工投产，力争完成技改投资250亿元以上。

做优园区企业。深入实施“2244”园区培育计划，支持各地园区走特色化、差异化、绿色化发展路子，力争工业集中度提高到74％。推进大企业发展、中小微企业成长工程，支持大企业大集团通过战略重组、投资扩能、市场化配置等方式增强实力，力争新增主营业务收入超50亿元、20亿元企业各1家，新培育规模以上工业企业50家。推动企业创新升级，建设智能车间、智能工厂。把握“去产能”宏观政策取向，统筹推进企业优胜劣汰，分类稳妥处置“僵尸企业”，有序引导富余产能转移，促进市场出清，化解过剩产能。实施质量对标提升行动，启动创建全国质量强市示范城市，力争硅产品质检中心通过国家验收。

稳定企业生产。开展降低实体经济企业成本行动，落实简政放权、减税降费等政策措施，促进企业降本增效。积极发展多层次资本市场，推动符合条件的企业利用主板、中小板、创业板、新三板等市场融资、交易，支持中小微企业利用直接债务融资，力争新增各类上市企业5家。建好主要工业品垂直电商平台，组织各类市场拓展活动，举办第二届陶博会、不锈钢产品交易博览会，提高“乐山造”产品市场份额。

四、坚持消费促进，着力壮大现代服务业

实施加快现代服务业发展行动，促进消费结构转型升级，实现服务业发展提速、比重提高、水平提升。

发展新兴服务业态。科学应对人口老龄化，大力发展健康养老产业，支持社会力量兴办养老健康服务机构，推进医养结合，加快构建以居家为基础、社区为依托、机构为补充的养老服务体系，满足多层次、个性化的健康养老服务需求。乘势发展电子商务，建成5个电子商务聚集区或创新孵化中心，确保电商交易额增长40％。加快四川金融次中心建设，支持市商业银行在“新三板”挂牌上市，鼓励发展社区银行、村镇银行等，创新发展互联网金融、普惠金融，筹建乐山农商行。做强现代物流业，抓好西南不锈钢第三方物流、市粮食物流园、棉竹电商快递园等项目建设。促进批发零售、住宿餐饮等传统服务业转型发展。

强化主体平台支撑。加大服务业重点企业、重点项目培育力度，争取更多企业和项目进入省级服务业“三百示范工程”培育库，确保新增服务业规模企业20家，服务业占经济总量的比重提高到31％。完善阿里巴巴、京东商城乐山地方馆电商平台建设，推进与携程、猪八戒、甲骨文等电商网络公司的合作，加快“全企入网”步伐。抓好四川物流公共信息平台乐山分平台建设，构建跨区域物流综合信息服务平台。发挥旅博会等会展平台作用，不断繁荣市场、营造商机、凝聚人气，促进会展经济健康发展。推进区域性服务业中心城市建设，争创2个省级服务业集聚区。

扩大多元消费需求。顺应消费结构升级趋势，加快推进消费品品质提升和供给创新，引导消费向智能、绿色、健康、安全方向转变。推进以满足新市民为出发点的住房制度改革，促进住房消费，稳定房地产市场。抓好国家信息消费试点和“三网融合”推广，引导广电、电信业务双向有序进入，拉动信息消费。积极培育文旅、教育、康养等消费热点，满足群众多样化消费需求。加大商品质量监管和消费维权力度，打造放心、舒心消费环境，保护消费者合法权益。

五、坚持夯实基础，着力发展现代农业

把坚持农民主体地位、增进农民福祉作为农村一切工作的出发点和落脚点，加快推进农业现代化，厚植农业农村发展优势，保持农业稳定发展和农民持续增收。

转变农业发展方式。深入推进粮食高产创建和粮经复合基地建设，新建高标准农田14万亩，稳定粮食总产量。扎实推进现代农业（林业、畜牧业）重点县建设，促进农产品加工业转型升级，确保农业产业化经营带动面再提高1个百分点。以生态有机为方向，抓好有机、绿色、无公害食品和农畜产品认证，加强农产品质量安全监管。加快现代茶叶基地建设，新（改）建茶园3万亩，办好首届茶博会，争创全国峨眉山茶产业知名品牌示范区。延伸农业功能和产业链，鼓励发展休闲体验、循环立体农业，提高农业综合效益。开展第三次全国农业普查。

建好幸福美丽新村。实施幸福美丽新村建设行动方案，新建彝家新寨28个、幸福美丽新村200个，新改建新村聚居点100个。创新农田水利建管运营模式，抓好小农水重点县、五小水利等项目，推进青衣江流域乐山灌区续建配套与节水改造。实施农村安全饮水巩固提升工程，新（改）建农村公路780公里，改造升级农村电网780公里，打通基础网络覆盖“最后一公里”。创新乡村治理，培育乡风文明，因地制宜加强新型农村社区和环境优美示范村庄建设，关爱帮扶农村留守儿童、妇女和老人。

促进农民持续增收。发挥多种形式农业适度规模经营引领作用，新培育家庭农场、专业大户、合作社、龙头企业等新型农业经营主体100个，有效组织农户对接大市场，稳定经营收益。深入实施乡村旅游提升工程和旅游富民工程，力争新创建省级乡村旅游强县1个、特色乡镇2个、精品特色乡村旅游经营点30个。做好农民企业家、新生代农民工、返乡农民工就业创业服务，激发农民创业创新活力，增加农民工资性收入。支持盘活农村资产资源，拓宽农民财产性收入来源。

六、坚持精准脱贫，着力补齐全面小康短板

按照“六个精准”“五个一批”要求，编制“十三五”扶贫专项规划，统筹推进连片与“插花”扶贫，实现精准脱贫3．5万人以上、贫困村“摘帽”不低于41个。

突出基础扶贫。统筹推进交通、水利、能源、通信等基础设施建设，加快改善贫困地区发展条件。实施小凉山交通建设三年行动方案和农村公路精准扶贫工程，开工建设峨汉高速、仁沐新高速等重大项目，完成贫困地区县乡道改善提升32公里、村道硬化87公里。推进金王寺水库枢纽渠系配套等水利工程建设，推动有条件的地方发展集中供水。鼓励发展太阳能、风能、沼气等清洁能源，解决贫困群众分散居住户用电用能问题。加快贫困地区宽带乡村、电视户户通、广播村村响等信息基础设施建设，缩小数字鸿沟，再消除225个行政村的宽带网络覆盖盲区。

强化产业扶贫。依托资源禀赋和比较优势，宜农则农、宜旅则旅、宜工则工，加快变资源优势为产业优势，不断增强造血扶贫功能。突出生态、绿色、安全，加快生态农业示范基地建设，提升贫困地区特色产业精深加工能力。鼓励发展“飞地园区”，推动互利共赢。积极推进旅游扶贫，打造彝族风情、生态观光、休闲度假等精品项目，创建一批旅游扶贫试验区、示范片和示范村。继续实施“一人就业、全家脱贫”行动，就地就近发展彝绣等致富产业。深入开展“互联网＋产业扶贫”行动，加快电子商务进农村步伐，促进“农产品进城”与“工业品下乡”双向流通。

推进内源扶贫。深入实施彝区“七个一”工程，提升公共服务水平，增强脱贫内生动力。抓好彝区“15年免费教育”“一村一支教”等行动，巩固“一村一幼”建设成果，新建彝区寄宿制学校10所。继续实施“远程医疗”“一村一卫”“一村一医”等工程，办好民族医士和幼师培训班。推进彝区“禁毒防艾”。强化社保政策兜底功能，加强农村低保和城乡居民养老保险、五保供养等社会救助制度的统筹衔接。坚持扶贫与扶志相结合，引导贫困地区群众移风易俗，发挥主观能动性，努力改变贫困落后面貌。整合用好扶贫项目资金，统筹各方帮扶力量，提升驻村帮扶实效。

七、坚持统筹协调，着力促进区域绿色发展

坚持问题导向，大力破解城乡区域发展不平衡、不协调、不可持续的问题，切实增强发展的整体性、协调性和可持续性。

加强生态环境保护。坚持绿色发展理念，深入实施主体功能区战略，合理划定城镇边界、生态空间和耕地红线，统筹推进聚集开发和分类保护。调整产业布局，支持重点开发区域布局发展战略性新兴产业、现代服务业和现代农业，鼓励限制开发区域大力发展特色农业、山地旅游和资源型、生态型产业。加强自然保护区、湿地、基本农田、城市绿心等保护管理，深入实施新一轮退耕还林。推进全国水生态文明城市试点，依法保护集中式饮用水源地，建成投运市第三污水处理厂。加强岷江、青衣江、大渡河等重点流域及其支流水环境防控，加大茫溪河、凌云河等小流域污染治理力度，守好长江上游生态屏障。加强大气、土壤、辐射环境及农村面源污染防治，做好灰霾等重污染天气联防联控。探索推进碳排放权交易、环境污染责任保险、企业环境信用评价等制度，促进经济、社会和生态效益相统一。

拓展区域发展空间。坚持以大交通促进大开放，加快建设成贵铁路、成昆铁路成峨段扩能工程、连乐铁路、绕城高速等项目，开工建设乐山机场、成昆铁路峨米段扩能工程等项目，力争开工成乐高速扩容改造项目，做好乐西高速等项目前期工作，推进“道口经济”发展，变“交通枢纽”为“经济枢纽”。加快建设井沙、峨夹、五犍沐等联网畅通工程，推进乐沙城际生态大道建设，完善与城镇体系相配套的公共交通体系，促进形成市区一体发展新格局。加强双边及多边合作，主动深化与“一带一路”沿线地区和各经济圈层的交流合作，密切与成都、成渝经济区及周边市州合作发展。积极申报设立乐山综合保税区，发展壮大半导体、化工、机电、鞋业等出口基地、外向型园区，鼓励优势企业走出去开拓市场、参与国际竞争，力争实现进出口总额9．3亿美元。

推进城乡一体发展。突出以人为核心的新型城镇化建设，推进“多规合一”和全域规划控制，守住景城相融、山水园林、文化传承等城市“基因”。打造特色城市新区，加快青江新区、临港新城、苏稽新区建设。合理发展城市组团，支持各地城市新区建设，积极实施“宜居县城建设行动”“百镇建设行动”，推进中心城市和县城、小城镇协调发展。强化城市基础设施建设，抓好嘉瑞大道、长青路、通棉路等道路新改建和大渡河大桥扩建、市综合垃圾处理场等项目，推进地下综合管廊建设试点。加快全国智慧城市试点建设，理顺市县城市管理体制，推广县级城管部门开展相对集中行政处罚权试点，促进城市精细化管理。推进城乡要素平等交换和公共资源均衡配置，增强基本公共服务覆盖常住人口能力。深化户籍制度改革，（紧转第3版）（紧接第2版）加快农业转移人口市民化进程，力争城镇化率提高1．5个百分点。

八、坚持民生为本，着力推进发展成果共享

从解决人民群众最关心最直接最现实的利益问题入手，坚守底线、突出重点、完善制度、引导预期，切实增进人民福祉，维护社会公平正义。

办好办实民生大事。深入实施十项民生工程，突出办好20件民生大事。加强就业创业平台建设，以创业带动就业，力争新增城镇就业3．8万人。实施全民参保计划，完善医疗保险政策，落实好城乡居民大病保险、临时救助、医疗救助等制度，适时调整最低工资、城乡低保、孤儿和农村五保对象供养标准。推进机关事业单位养老保险制度改革。深化“百万安居工程建设行动”，提高棚户区改造货币化安置率。做好住房地震保险试点。开工建设市残疾人综合服务中心，完成县级残疾人康复托养服务中心建设。启动实施“十三五”妇女儿童事业发展规划，深化未成年人社会保护试点。积极发展慈善事业。做好对口支援理塘工作。

加大公共服务供给。促进教育均衡发展，改善农村义务教育薄弱学校基本办学条件，加快乐山一中、市实验中学、乐师附小、乐山八中等新改建项目。完成乐山职业技术学院新校区主体工程建设，抓好中职学校办学达标工程。加强特殊教育、民办教育，启动四川特殊教育职业技术学院建设。推进基本医疗卫生服务均等化，巩固完善县级公立医院综合改革和分级诊疗制度。抓好市儿童专科医院等项目建设。鼓励发展社会办医。加强重大疾病防控和职业病危害防治。完善计生服务管理，实施好全面两孩政策。构建现代公共文化和体育服务体系，推进国家公共文化服务体系示范区建设，启动市图书馆改扩建，新建市级以上全民健身工程200个。推进数字档案馆建设。提升气象现代化水平。做好邮政、外侨、人防、对台、保密、地方志等工作。

提升社会治理水平。深入践行社会主义核心价值观，创建省级文明城市。深化“法律七进”活动，启动“七五”普法。创新社区治理方式，建好社区公共服务综合信息平台，做好第十届村（居）委会换届选举。整合人民调解、行政调解、司法调解作用，依法规范信访秩序，化解社会矛盾。提升依法治网水平，营造良好网络环境。抓好基层公安机关营区化建设，编织全方位、立体化公共安全网，依法打击各类违法犯罪活动。完善安全生产责任体系和隐患排查治理体系，推进监管信息平台建设，促进“打非治违”常态化。创新食品药品安全监管模式，推动社会共治。分级完善应急平台体系，构建“平战一体”的应急管理模式，提升突发事件预防预警和应急处置能力。统筹抓好国防教育、国防动员和后备力量建设。妥善做好移民安置，巩固移民后期扶持成果。做好新形势下民族宗教工作。

九、坚持转变职能，着力加强政府自身建设

巩固“三严三实”专题教育成果，始终保持过硬的政治定力、思想定力、作风定力，推动法治、创新、廉洁和服务型政府建设取得新的更大进展。

坚持依法行政。深入实施法治政府建设实施纲要，加快建设职能科学、权责法定、执法严明、公开公正、廉洁高效、守法诚信的法治政府。深化行政审批制度改革，公布市县两级行政权力清单、责任清单。健全依法决策机制，加强合法性审查，增强公众参与、专家论证和风险评估实效。实行重大决策终身责任追究及责任倒查机制，提高决策公信力和执行力。抓好全国综合行政执法体制改革试点，统筹归并执法机构和队伍种类，支持在文化旅游、公共安全、城乡建设、市场监管等领域推行跨部门综合执法，启动环保机构监测监察执法垂直管理。完善行政执法程序，建立行政执法信息平台和监督网络平台，强化行政执法人员和执法辅助人员管理，保障严格规范公正文明执法。

坚持从严治政。把纪律建设摆在更加突出位置，坚持纪严于法、纪在法前，始终用纪律管住管好干部队伍。持之以恒落实中央八项规定和省市十项规定精神，驰而不息整治“四风”顽疾，把作风建设抓到底。完善政务督查、效能监察、行政约谈等制度，加大纠错问责追责力度，做到有责必问、问责必严，坚决惩治庸政懒政怠政，坚决抵制各种歪风邪气，促进党风政风民风向善向上。启动市政务服务国家级标准化试点工作，抓好政务服务云平台、便民服务平台、公共资源交易平台等建设。完善政务公开、新闻发言人、突发事件信息发布等制度，健全行政权力依法规范公开运行机制，打造阳光政务。

坚持廉洁从政。严格落实“两个责任”，加强权力运行监督制约，进一步扎紧用权管权限权的制度“围栏”。对财政资金分配使用、国有资产监管、政府投资与采购、公共资源转让、公共工程建设等权力集中部门和岗位实行分事行权、分岗设权、分级授权，定期轮岗。完善政府内部层级监督，加大审计监督、财政监督和监察监督力度，强化“三公”经费预算管理，完成公务用车制度改革。主动接受市人大及其常委会法律监督、工作监督和市政协民主监督，广泛听取民主党派、无党派人士、工商联、人民团体和社会各界的意见建议，自觉接受人民监督。坚持紧抓不放、利剑高悬，支持执纪执法机关查处行政案件和违纪违法行为，做到惩治腐败无禁区、全覆盖、零容忍。

各位代表：站在新起点、适应新常态、更需新作为。让我们在省委、省政府和市委的坚强领导下，紧紧依靠全市人民，脚踏实地、苦干实干、砥砺前行，奋力开创“十三五”发展新局面，为乐山转型升级、美丽发展、全面建成小康社会而不懈奋斗！
